# The prevalence of statistical reporting errors in psychology (1985–2013)

**DOI:** 10.3758/s13428-015-0664-2

**Published:** 2015-10-23

**Authors:** Michèle B. Nuijten, Chris H. J. Hartgerink, Marcel A. L. M. van Assen, Sacha Epskamp, Jelte M. Wicherts

**Affiliations:** 1 Department of Methodology and Statistics, Tilburg School of Social and Behavioral Sciences, Tilburg University, Tilburg, Netherlands; 2 Psychological Methods, University of Amsterdam, Amsterdam, Netherlands

**Keywords:** Reporting errors, *p*-values, Significance, False positives, NHST, Questionable research practices, Publication bias

## Abstract

This study documents reporting errors in a sample of over 250,000 *p*-values reported in eight major psychology journals from 1985 until 2013, using the new R package “statcheck.” statcheck retrieved null-hypothesis significance testing (NHST) results from over half of the articles from this period. In line with earlier research, we found that half of all published psychology papers that use NHST contained at least one *p*-value that was inconsistent with its test statistic and degrees of freedom. One in eight papers contained a grossly inconsistent *p*-value that may have affected the statistical conclusion. In contrast to earlier findings, we found that the average prevalence of inconsistent *p*-values has been stable over the years or has declined. The prevalence of gross inconsistencies was higher in *p*-values reported as significant than in *p*-values reported as nonsignificant. This could indicate a systematic bias in favor of significant results. Possible solutions for the high prevalence of reporting inconsistencies could be to encourage sharing data, to let co-authors check results in a so-called “co-pilot model,” and to use statcheck to flag possible inconsistencies in one’s own manuscript or during the review process.

Most conclusions in psychology are based on the results of null hypothesis significance testing (NHST; Cumming et al., 2007; Hubbard & Ryan, 2000; Sterling, 1959; Sterling, Rosenbaum, & Weinkam, 1995). Therefore, it is important that NHST is performed correctly and that NHST results are reported accurately. However, there is evidence that many reported *p*-values do not match their accompanying test statistic and degrees of freedom (Bakker & Wicherts, 2011; Bakker & Wicherts, 2014; Berle & Starcevic, 2007; Caperos & Pardo, 2013; Garcia-Berthou & Alcaraz, 2004; Veldkamp, Nuijten, Dominguez-Alvarez, Van Assen, & Wicherts, 2014; Wicherts, Bakker, & Molenaar, 2011). These studies highlighted that roughly half of all published empirical psychology articles using NHST contained at least one inconsistent *p*-value and that around one in seven articles contained a gross inconsistency, in which the reported *p*-value was significant and the computed *p*-value was not, or vice versa.

This alarmingly high error rate can have large consequences. Reporting inconsistencies could affect whether an effect is perceived to be significant or not, which can influence substantive conclusions. If a result is inconsistent it is often impossible (in the absence of raw data) to determine whether the test statistic, the degrees of freedom, or the *p*-value were incorrectly reported. If the test statistic is incorrect and it is used to calculate the effect size for a meta-analysis, this effect size will be incorrect as well, which could affect the outcome of the meta-analysis (Bakker & Wicherts, 2011; in fact, the misreporting of all kinds of statistics is a problem for meta-analyses; Gotzsche, Hrobjartsson, Maric, & Tendal, 2007; Levine & Hullett, 2002). Incorrect *p*-values could affect the outcome of tests that analyze the distribution of *p*-values, such as the *p*-curve (Simonsohn, Nelson, & Simmons, 2014) and *p*-uniform (Van Assen, Van Aert, & Wicherts, 2014). Moreover, Wicherts et al. (2011) reported that a higher prevalence of reporting errors were associated with a failure to share data upon request.

Even though reporting inconsistencies can be honest mistakes, they have also been categorized as one of several fairly common questionable research practices (QRPs) in psychology (John, Loewenstein, & Prelec, 2012). Interestingly, psychologists’ responses to John et al.’s survey fitted a Guttman scale reasonably well. This suggests that a psychologist’s admission to a QRP that is less often admitted to by others usually implies his or her admission to QRPs with a higher admission rate in the entire sample. Given that rounding down *p*-values close to .05 was one of the QRPs with relatively low admission rates, the frequency of misreported *p*-values could provide information on the frequency of the use of more common QRPs. The results of John et al. would therefore imply that a high prevalence of reporting errors (or more specifically, incorrect rounding down of *p*-values to be below .05) can be seen as an indicator of the use of other QRPs, such as the failure to report all dependent variables, collecting of more data after seeing whether results are significant, failing to report all conditions, and stopping data collection after achieving the desired result. Contrary to many other QRPs in John et al.’s list, misreported *p*-values that bear on significance can be readily detected on the basis of the articles’ text.

Previous research found a decrease in negative results (Fanelli, 2012) and an increase in reporting inconsistencies (Leggett, Thomas, Loetscher, & Nicholls, 2013), suggesting that QRPs are on the rise. On the other hand, it has been found that the number of published corrections to the literature did not change over time, suggesting no change in QRPs over time (Fanelli, 2013, 2014). Studying the prevalence of misreported *p*-values over time could shed light on possible changes in the prevalence of QRPs.

Besides possible changes in QRPs over time, some evidence suggests that the prevalence of QRPs may differ between subfields of psychology. Leggett et al. (2013) recently studied reporting errors in two main psychology journals in 1965 and 2005. They found that the increase in reporting inconsistencies over the years was higher in the *Journal of Personality and Social Psychology* (JPSP), the flagship journal of social psychology, than in the *Journal of Experimental Psychology: General* (JEPG). This is in line with the finding of John et al. (2012) that social psychologists admit to more QRPs, find them more applicable to their field, and find them more defensible as compared to other subgroups in psychology (but see also Fiedler & Schwarz, 2015, on this issue). However, the number of journals and test results in Leggett et al.’s study was rather limited and so it is worthwhile to consider more data before drawing conclusions with respect to differences in QRPs between subfields in psychology.

The current evidence for reporting inconsistencies is based on relatively small sample sizes of articles and *p*-values. The goal of our current study was to evaluate reporting errors in a large sample of more than a quarter million *p*-values retrieved from eight flagship journals covering the major subfields in psychology. Manually checking errors is time-consuming work, therefore we present and validate an automated procedure in the R package *statcheck* (Epskamp & Nuijten, 2015). The validation of statcheck is described in Appendix A.

We used statcheck to investigate the overall prevalence of reporting inconsistencies and compare our findings to findings in previous studies. Furthermore, we investigated whether there has been an increase in inconsistencies over the period 1985 to 2013, and, on a related note, whether there has been any increase in the number of NHST results in general and per paper. We also documented any differences in the prevalence and increase of reporting errors between journals. Specifically, we studied whether articles in social psychology contain more inconsistencies than articles in other subfields of psychology.

## Method

### “statcheck”

To evaluate the prevalence of reporting errors, we used the automated procedure *statcheck* (version 1.0.1.; Epskamp & Nuijten, 2015). This freely available R package (R Core Team, 2014) extracts statistical results and recalculates *p*-values based on reported test statistics and their degrees of freedom. Roughly, the underlying procedure executes the following four steps.

- *Step 1:* First, statcheck converts a PDF or HTML file to a plain text file. The conversion from PDF to plain text can sometimes be problematic, because some journal publishers use images of signs such as “<”, “>”, or “=”, instead of the actual character. These images are not converted to the text file. HTML files do not have such problems and typically render accurate plain text files.
- *Step 2:* From the plain text file, statcheck extracts *t*, *F*, *r*, χ^2^, and *Z* statistics, with the accompanying degrees of freedom (*df*) and *p*-value. Since statcheck is an automated procedure, it can only search for prespecified strings of text. Therefore, we chose to let statcheck search for results that are reported completely and exactly in APA style (American Psychological Association, 2010). A general example would be “*test statistic* (df1, df2) =/</> …, *p* =/</> …”. Two more specific examples are: “*t*(37) = −4.93, *p* <.001”, “*χ* ^2^(1, N = 226) = 6.90, *p* <.01.” statcheck takes different spacing into account, and also reads results that are reported as nonsignificant (*ns*). On the other hand, it does not read results that deviate from the APA template. For instance, statcheck overlooks cases in which a result includes an effect size estimate in between the test statistic and the *p*-value (e.g., “*F*(2, 70) = 4.48, *MSE* = 6.61, *p* <.02”) or when two results are combined into one sentence (e.g., “*F*(1, 15) = 19.9 and 5.16, *p* <.001 and *p* <.05, respectively”). These restrictions usually also imply that statcheck will not read results in tables, since these are often incompletely reported (see Appendix A for a more detailed overview of what statcheck can and cannot read).
- *Step 3:* statcheck uses the extracted test statistics and degrees of freedom to recalculate the *p*-value. By default all tests are assumed to be two-tailed. We compared *p*-values recalculated by statcheck in R version 3.1.2 and Microsoft Office Excel 2013 and found that the results of both programs were consistent up to the tenth decimal point. This indicates that underlying algorithms used to approximate the distributions are not specific to the R environment.
- *Step 4:* Finally, statcheck compares the reported and recalculated *p*-value. Whenever the reported *p*-value is inconsistent with the recalculated *p*-value, the result is marked as an *inconsistency*. If the reported *p*-value is inconsistent with the recalculated *p*-value and the inconsistency changes the statistical conclusion (assuming α = .05), the result is marked as a *gross inconsistency*. To take into account one-sided tests, statcheck scans the whole text of the article for the words “one-tailed,” “one-sided,” or “directional.” If a result is initially marked as inconsistent, but the article mentions one of these words *and* the result would have been consistent if it were one-sided, then the result is marked as consistent. Note that statcheck does not take into account *p*-values that are adjusted for multiple testing (e.g., a Bonferroni correction). *P*-values adjusted for multiple comparisons that are higher than the recalculated *p*-value can therefore erroneously be marked as inconsistent. However, when we automatically searched our sample of 30,717 articles, we found that only 96 articles reported the string “Bonferroni” (0.3 %) and nine articles reported the string “Huynh-Feldt” or “Huynh Feldt” (0.03 %). We conclude from this that corrections for multiple testing are rarely used and will not significantly distort conclusions in our study.

Similar to Bakker and Wicherts (2011), statcheck takes numeric rounding into account. Consider the following example: *t*(28) = 2.0, *p*<.05. The recalculated *p*-value that corresponds to a *t*-value of 2.0 with 28 degrees of freedom is .055, which appears to be inconsistent with the reported *p*-value of <.05. However, a reported *t*-value of 2.0 could correspond to any rounded value between 1.95 and 2.05, with a corresponding range of *p*-values between .0498 and .0613, which means that the reported *p* <.05 is not considered inconsistent.

Furthermore, statcheck considers *p*-values reported as *p* = .05 as significant. We inspected 10 % of the 2,473 instances in our sample in which a result was reported as “*p* = .05” and inspected whether these *p*-values were interpreted as significant. In the cases where multiple *p*-values from the same article were selected, we only included the *p*-value that was drawn first to avoid dependencies in the data. Our final sample consisted of 236 instances where “*p* = .05” was reported and of these *p*-values 94.3 % was interpreted as being significant. We therefore decided to count *p*-values reported as “*p* = .05” as indicating that the authors presented the result as significant.

The main advantage of statcheck is that it enables searching for reporting errors in very large samples, which would be infeasible by hand. Furthermore, manual checking is subject to human error, which statcheck eliminates. The disadvantage of statcheck is that it is not as comprehensive as a manual procedure, because it will miss results that deviate from standard reporting and results in tables, and it does not take into account adjustments on *p*-values. Consequently, statcheck will miss some reported results and will incorrectly earmark some correct *p*-values as a reporting error. Even though it is not feasible to create an automated procedure that is as accurate as a manual search in veryfying correctness of the results, it is important to exclude the possibility that statcheck yields a biased depiction of the true inconsistency rate. To avoid bias in the prevalence of reporting errors, we performed a validity study of statcheck, in which we compared statcheck’s results with the results of Wicherts, Bakker, and Molenaar (2011), who performed a manual search for and verification of reporting errors in a sample of 49 articles.

The validity study showed that statcheck read 67.5 % of the results that were manually extracted. Most of the results that statcheck missed were either reported with an effect size between the test statistics and the *p*-value (e.g., *F*(2, 70) = 4.48, MSE = 6.61, *p* <.02; 201 instances in total) or reported in a table (150 instances in total). Furthermore, Wicherts et al. found that 49 of 1,148 *p*-values were inconsistent (4.3 %) and ten of 1,148 *p*-values were grossly inconsistent (.9 %), whereas statcheck (with automatic one-tailed test detection) found that 56 of 775 *p*-values were inconsistent (7.2 %) and eight of 775 *p*-values were grossly inconsistent (1.0 %). The higher inconsistency rate found by statcheck was mainly due to our decision to count *p* = .000 as incorrect (a *p*-value cannot exactly be zero), whereas this was counted correct by Wicherts et al. If we do not include these 11 inconsistencies due to *p* = .000, statcheck finds an inconsistency percentage of 5.8 % (45 of 775 results), 1.5 percentage points higher than in Wicherts et al. This difference was due to the fact that statcheck did not take into account 11 corrections for multiple testing and Wicherts et al. did. The inter-rater reliability in this scenario between the manual coding in Wicherts et al. and the automatic coding in statcheck was .76 for the inconsistencies and .89 for the gross inconsistencies. Since statcheck slightly overestimated the prevalence of inconsistencies in this sample of papers, we conclude that statcheck can render slightly different inconsistency rates than a search by hand. Therefore, the results of statcheck should be interpreted with care. For details of the validity study and an explanation of all discrepancies between statcheck and Wicherts et al., see Appendix A.

### Sample

A pilot study of social science journals in the Web of Science citation data base showed that few journals outside psychology include APA reporting style, therefore we limited our sample to psychology journals. As explained above, statcheck cannot always read results from articles in PDF due to problems in the conversion from PDF to plain text. These problems do not occur in articles in HTML format. Therefore, to obtain the most reliable statcheck results we restricted our sample to articles that were available in HTML format. The time span over which we downloaded articles depended on the year a journal started to publish articles in HTML. We collected the data in 2014, so we included articles up until 2013 to ensure complete sets of articles for an entire year. Via EBSCOhost we manually downloaded all articles in HTML from 1985 to 2013 from six flagship psychology journals that represent six main sub disciplines: *Journal of Applied Psychology* (JAP; Applied Psychology), *Journal of Consulting and Clinical Psychology* (JCCP; Clinical Psychology), *Developmental Psychology* (DP; Developmental Psychology), *Journal of Experimental Psychology: General* (JEPG; Experimental Psychology), and *Journal of Personality and Social Psychology* (JPSP; Social Psychology). These journals are published by the APA and follow the APA reporting guidelines. Furthermore, we manually downloaded all articles in HTML from two journals in general psychology: *Psychological Science* (PS; 2003–2013) and *Frontiers in Psychology* (FP; 2010–2013). In this manual download we did not include retractions, errata, and editorials. Finally, we automatically downloaded all HTML articles with the subject “psychology” from the *Public Library of Science* (PLoS; 2000–2013), using the rplos R package (Chamberlain, Boettiger, & Ram, 2014).^1^ In this automatic process we did not exclude retractions, errata, or editorials. The final sample consisted of 30,717 articles. The number of downloaded articles per journal is given in Table 1. To obtain reporting error prevalences for each subfield and for psychology in total, statcheck was used on all downloaded articles.

**Table 1:** Specifications of the years from which HTML articles were available, the number of downloaded articles per journal, the number of articles with APA-reported null-hypothesis significance testing (NHST) results, the number of APA-reported NHST results, and the median number of APA-reported NHST results per article

Journal	Subfield	Years included	No. of articles	No. of articles with NHST results		No. of NHST results	Median no. of NHST results per article with NHST results
PLOS	General	2000-2013	10,299	2,487	(24.1 %)	31,539	9
JPSP	Social	1985-2013	5,108	4,346	(85.1 %)	101,621	19
JCCP	Clinical	1985-2013	3,519	2,413	(68.6 %)	27,429	8
DP	Developmental	1985-2013	3,379	2,607	(77.2 %)	37,658	11
JAP	Applied	1985-2013	2,782	1,638	(58.9 %)	15,134	6
PS	General	2003-2013	2,307	1,681	(72.9 %)	15,654	8
FP	General	2010-2013	2,139	702	(32.8 %)	10,149	10
JEPG	Experimental	1985-2013	1,184	821	(69.3 %)	18,921	17
Total			30,717	16,695	(54.4%)	258,105	11

### Statistical analyses

Our population of interest is all APA-reported NHST results in the full text of the articles from the eight selected flagship journals in psychology from 1985 until 2013. Our sample includes this entire population. We therefore made no use of inferential statistics, since inferential statistics are only necessary to draw conclusions about populations when having much smaller samples. We restricted ourselves to descriptive statistics; every documented difference or trend entails a difference between or trend in the entire population or subpopulations based on journals. For linear trends we report regression weights and percentages of variance explained to aid interpretation.

## Results

We report the prevalence of reporting inconsistencies at different levels. We document general prevalence of NHST results and present percentages of articles that use NHST per journal and over the years. Because only the five APA journals provided HTMLs for all years from 1985 to 2013, the overall trends are reported for APA journals only, and do not include results from Psychological Science, PLoS, and Frontiers, which only cover recent years. Reporting inconsistencies are presented both at the level of article and at the level of the individual *p*-value, i.e., the percentage of articles with at least one inconsistency and the average percentage of *p*-values within an article that is inconsistent, respectively. We also describe differences between journals and trends over time.

### Percentage of articles with null-hypothesis significance testing (NHST) results

**Fig. 1:**
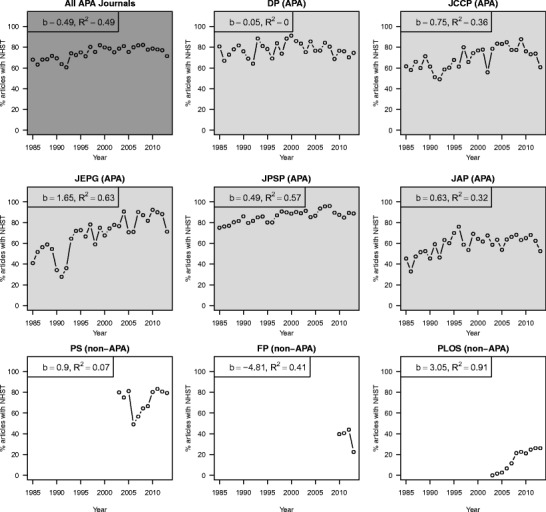
The percentage of articles with American Psychological Association (APA)-reported null-hypothesis significance testing (NHST) results over the years, averaged over all APA journals (*Developmental Psychology* (DP), *Journal of Consulting and Clinical Psychology* (JCCP), *Journal of Experimental Psychology: General* (JEPG), *Journal of Personality and Social Psychology* (JPSP), and *Journal of Applied Psychology* (JAP); dark gray panel), and split up per journal – light gray panels for the APA journals and white panels for the non-APA journals (*Psychological Science* (PS), *Frontiers in Psychology* (FP), and *Public Library of Science* (PLoS)). For each trend we report the unstandardized linear regression coefficient (b) and the coefficient of determination (R^2^) of the linear trend

Overall, statcheck detected NHST results in 54.4 % of the articles, but this percentage differed per journal. The percentage of articles with at least one detected NHST result ranged from 24.1 % in PLoS to 85.1 % in JPSP (see Table 1). This can reflect a difference in the number of null-hypothesis significance tests performed, but it could also reflect a difference in the rigor with which the APA reporting standards are followed or how often tables are used to report results. Figure 1 shows the percentage of downloaded articles that contained NHST results over the years, averaged over all APA journals (DP, JCCP, JEPG, JPSP, and JAP; dark gray panel), and split up per journal (light gray panels for the APA journals and white panels for the non-APA journals). All journals showed an increase in the percentage of articles with APA-reported NHST results over the years except for DP and FP, for which this rate remained constant and/or declined, respectively. Appendix B lists the number of articles with NSHT results over the years per journal.

### Number of published NHST results over the years

**Fig. 2:**
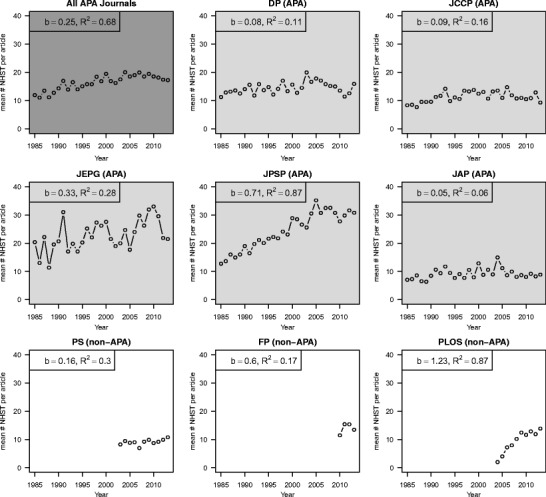
The average number of American Psychological Association (APA)-reported null-hypothesis significance testing (NHST) results per article that contains NHST results over the years, averaged over all APA journals (*Developmental Psychology* (DP), *Journal of Consulting and Clinical Psychology* (JCCP), *Journal of Experimental Psychology: General* (JEPG), *Journal of Personality and Social Psychology* (JPSP), and *Journal of Applied Psychology* (JAP); dark gray panel), and split up per journal (light gray panels for the APA journals and white panels for the non-APA journals – *Psychological Science* (PS), *Frontiers in Psychology* (FP), and *Public Library of Science* (PLoS)). For each trend we report the unstandardized linear regression coefficient (b) and the coefficient of determination (R^2^) of the linear trend

We inspected the development of the average number of APA-reported NHST results per article, given that the article contained at least one detectable NHST result (see Fig. 2). Note that in 1985 the APA manual already required statistics to be reported in the manner that statcheck can read (American Psychological Association, 1983). Hence, any change in retrieved NHST results over time should reflect the actual change in the number of NHST results reported in articles rather than any change in the capability of statcheck to detect results.

Across all APA journals, the number of NHST results per article has increased over the period of 29 years (b = .25, R^2^ = .68), with the strongest increases in JEPG and JPSP. These journals went from an average of around 10–15 NHST results per article in 1985 to as much as around 30 results per article on average in 2013. The mean number of NHST results per article remained relatively stable in DP, JCCP, and JAP; over the years, the articles with NHST results in these journals contained an average of ten NHST results. It is hard to say anything definite about trends in PS, FP, and PLOS, since we have only a limited number of years for these journals (the earliest years we have information for are 2003, 2010, and 2004, respectively). Both the increase in the percentage of articles that report NHST results and the increased number of NHST results per article show that NHST is increasingly popular in psychology. It is therefore important that the results of these tests are reported correctly.

### General prevalence of inconsistencies

Across all journals and years 49.6 % of the articles with NHST results contained at least one inconsistency (8,273 of the 16,695 articles) and 12.9 % (2,150) of the articles with NHST results contained at least one gross inconsistency. Furthermore, overall 9.7 % (24,961) of the *p*-values were inconsistent, and 1.4 % (3,581) of the *p*-values were grossly inconsistent. We also calculated the percentage of inconsistencies per article and averaged these percentages over all articles. We call this the “(gross) inconsistency rate.” Across journals, the inconsistency rate was 10.6 % and the gross inconsistency rate was 1.6 %.

### Prevalence of inconsistencies per journal

**Fig. 3:**
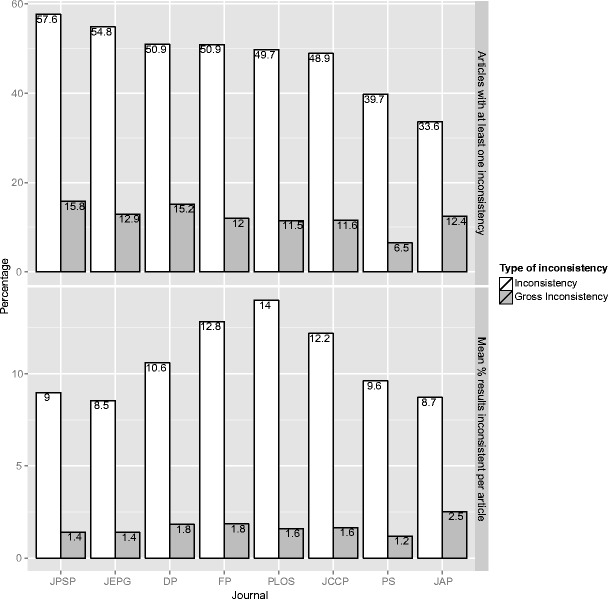
The average percentage of articles within a journal with at least one (gross) inconsistency and the average percentage of (grossly) inconsistent *p*-values per article, split up by journal. Inconsistencies are depicted in white and gross inconsistencies in grey. For the journals *Journal of Personality and Social Psychology* (JPSP), *Journal of Experimental Psychology: General* (JEPG), *Developmental Psychology* (DP), *Frontiers in Psychology* (FP), *Public Library of Science* (PLoS), *Journal of Consulting and Clinical Psychology* (JCCP), *Psychological Science* (PS), and *Journal of Applied Psychology* (JAP), respectively, the number of articles with null-hypothesis significance testing (NHST) results is 4,346, 821, 2,607, 702, 2,487, 2,413, 1,681, and 1,638, and the average number of NHST results in an article is 23.4, 23.0, 14.4, 14.5, 12.7, 11.4, 9.3, and 9.2

We calculated the prevalence of inconsistencies per journal at two levels. First, we calculated the percentage of articles with NHST results per journal that contained at least one (gross) inconsistency. Second, we calculated the inconsistency rate per journal. The top panel of Fig. 3 shows the average percentage of articles with at least one (gross) inconsistency, per journal. The journals are ordered from the journal with the highest percentage of articles with an inconsistency to the journal with the least articles with an inconsistency. JPSP showed the highest prevalence of articles with at least one inconsistency (57.6 %), followed by JEPG (54.8 %). The journals in which the percentage of articles with an inconsistency was lowest are PS and JAP (39.7 % and 33.6 % respectively). JPSP also had the highest percentage of articles with at least one gross inconsistency (15.8 %), this time followed by DP (15.2 %). PS had the lowest percentage of articles with gross inconsistencies (6.5 %).

The inconsistency rate shows a different pattern than the percentage of articles with all inconsistencies. PLoS showed the highest percentage of inconsistent *p*-values per article overall, followed by FP (14.0 % and 12.8 %, respectively). Furthermore, whereas JPSP was the journal with the highest percentage of articles with inconsistencies, it had one of the lowest probabilities that a *p*-value in an article was inconsistent (9.0 %). This discrepancy is caused by a difference between journals in the number of *p*-values per article: the articles in JPSP contain many *p*-values (see Table 1, right column). Hence, notwithstanding a low probability of a single *p*-value in an article being inconsistent, the probability that an article contained at least one inconsistent *p*-value was relatively high. The gross inconsistency rate was quite similar over all journals except JAP, in which the gross inconsistency rate was relatively high (2.5 %).

### Prevalence of inconsistencies over the years

**Fig. 4:**
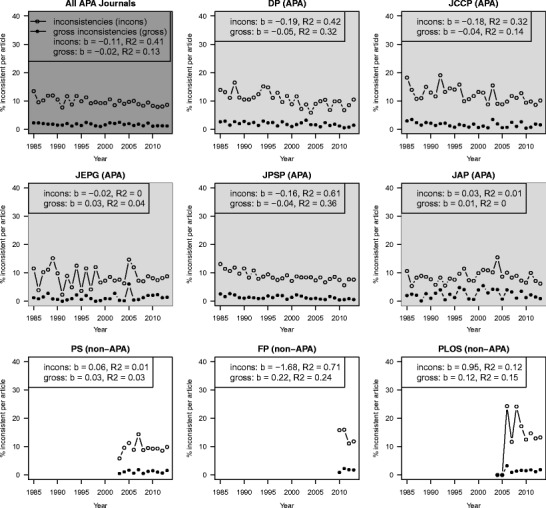
Average percentage of inconsistencies (open circles) and gross inconsistencies (solid circles) in an article over the years averaged over all American Psychological Association (APA) journals (*Developmental Psychology* (DP), *Journal of Consulting and Clinical Psychology* (JCCP), *Journal of Experimental Psychology: General* (JEPG), *Journal of Personality and Social Psychology* (JPSP), and *Journal of Applied Psychology* (JAP); dark gray panel) and split up per journal (light gray panels for the APA journals and white panels for non-APA journals – *Psychological Science* (PS), *Frontiers in Psychology* (FP), and *Public Library of Science* (PLoS)). The unstandardized regression coefficient b and the coefficient of determination R^2^ of the linear trend are shown per journal for both inconsistencies (incons) and gross inconsistencies (gross) over the years

If gross inconsistencies are indicative of QRPs and QRPs have increased over the years, we would expect an increase of gross inconsistencies over the years (see also Leggett et al., 2013). To study this, we inspected the gross inconsistency rate in journals over time. The results are shown in Fig. 4.

The number of (gross) inconstencies has decreased or remained stable over the years across the APA journals. In DP, JCCP, JPEG, and JPSP the percentage of all inconsistencies in an article has decreased over the years. For JAP there is a positive (but very small) regression coefficient for year, indicating an increasing error rate, but the R^2^ is close to zero. The same pattern held for the prevalence of gross inconsistencies over the years. DP, JCCP, and JPSP have shown a decrease in gross inconsistencies, in JEPG and JAP the R^2^ is very small, and the prevalence seems to have remained practically stable. The trends for PS, FP, and PLoS are hard to interpret given the limited number of years of covarage. Overall, it seems that, contrary to the evidence suggesting that the use of QRPs could be on the rise (Fanelli, 2012; Leggett et al., 2013), neither the inconsistencies nor the gross inconsistencies have shown an increase over time. If anything, the current results reflect a decrease of reporting error prevalences over the years.

**Fig. 5:**
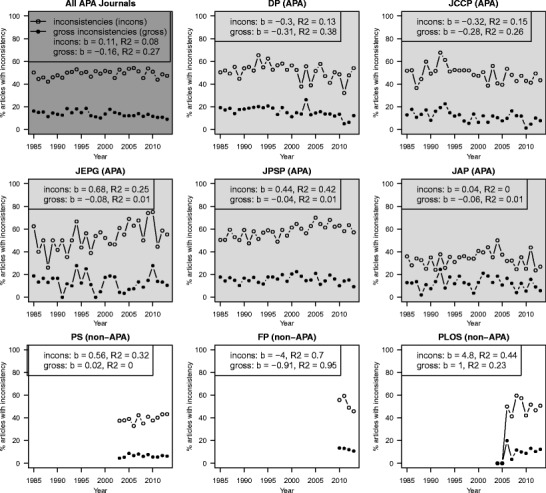
Percentage of articles with at least one inconsistency (open circles) or at least one gross inconsistency (solid circles), split up by journal. The unstandardized regression coefficient b and the coefficient of determination R^2^ of the linear trend are shown per journal for both inconsistencies (incons) as gross inconsistencies (gross) over the years. *APA* American Psychological Assocation, *DP* Developmental Psychology, *JCCP* Journal of Consulting and Clinical Psychology, JEPG Journal of Experimental Psychology: General , *JPSP* Journal of Personality and Social Psychology, *JAP* Journal of Applied Psychology, *PS* Psychological Science, *FP* Frontiers in Psychology, *PLoS* Public Library of Science

We also looked at the development of inconsistencies at the article level. More specifically, we looked at the percentage of articles with at least one inconsistency over the years, averaged over all APA journals (DP, JCCP, JEPG, JPSP, and JAP; dark gray panel in Fig. 5) and split up per journal (light gray panels for the APA journals and white panels for the non-APA journals in Fig. 5). Results show that there has been an increase in JEPG and JPSP for the percentage of articles with NHST results that have at least one inconsistency, which is again associated with the increase in the number of NHST results per article in these journals (see Fig. 2). In DP and JCCP, there was a decrease in articles with an inconsistency. For JAP there is no clear trend; the R^2^ is close to zero. A more general trend is evident in the prevalence of articles with gross inconsistencies: in all journals, except PS and PLOS, the percentage of articles with NHST that contain at least one gross inconsistency has been decreasing. Note that the trends for PS, FP, and PLOS are unstable due to the limited number of years we have data for. Overall, it seems that, even though the prevalence of articles with inconsistencies has increased in some journals, the prevalence of articles with gross inconsistencies has shown a decline over the studied period.

### Prevalence of gross inconsistencies in results reported as significant and nonsignificant

**Fig. 6:**
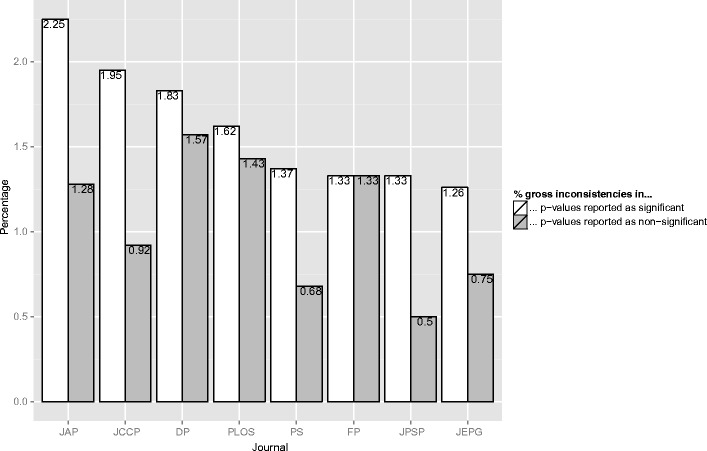
The percentage of gross inconsistencies in *p*-values reported as significant (white bars) and nonsignificant (gray bars), split up by journal. For the journals *Journal of Applied Psychology* (JAP), *Journal of Consulting and Clinical Psychology* (JCCP), *Developmental Psychology* (DP), *Public Library of Science* (PLoS), *Psychological Science* (PS), *Frontiers in Psychology* (FP), *Journal of Personality and Social Psychology* (JPSP), and *Journal of Experimental Psychology: General* (JEPG), respectively, the total number of significant *p*-values was 11,654, 21,120, 29,962, 22,071, 12,482, 7,377, 78,889, and 14,084, and the total number of nonsignificant *p*-values was 3,119, 5,558, 6,698, 9,134, 2,936, 2,712, 17,868, and 4,407

We inspected the gross inconsistencies in more detail by comparing the percentage of gross inconsistencies in *p*-values reported as significant and *p*-values reported as nonsignificant. Of all *p*-values reported as significant 1.56 % was grossly inconsistent, whereas only .97 % of all *p*-values reported as nonsignificant was grossly inconsistent, indicating it is more likely for a *p*-value reported as significant to be a gross inconsistency than for a *p*-value reported as nonsignificant. We also inspected the prevalence of gross inconsistencies in significant and non-significant *p*-values per journal (see Fig. 6). In all journals, the prevalence of gross inconsistencies is higher in significant *p*-values than in nonsignificant *p*-values (except for FP, in which the prevalence is equal in the two types of *p*-values). This difference in prevalence is highest in JCCP (1.03 percentage point), JAP (.97 percentage point), and JPSP (.83 percentage point) respectively, followed by JEPG (.51 percentage point) and DP (.26 percentage point), and smallest in PLOS (.19 percentage point) and FP (.00 percentage point).

It is hard to interpret the percentages of inconsistencies in significant and nonsignificant *p*-values substantively, since they depend on several factors, such as the specific *p*-value: it seems more likely that a *p*-value of .06 is reported as smaller than .05 than a *p*-value of .78. That is, because journals may differ in the distribution of specific *p*-values we should also be careful in comparing gross inconsistencies in *p*-values reported as significant across journals. Furthermore, without the raw data it is impossible to determine whether it is the *p*-value that is erroneous, or the test statistic or degrees of freedom. As an example of the latter case, a simple typographical error such as “*F*(2,56) = 1.203, *p* < .001” instead of “*F*(2,56) = 12.03, *p* < .001” produces a gross inconsistency, without the *p*-value being incorrect. Although we cannot interpret the absolute percentages and their differences, the finding that gross inconsistencies are more likely in *p*-values presented as significant than in *p*-values presented as nonsignificant could indicate a systematic bias and is reason for concern.

**Fig. 7:**
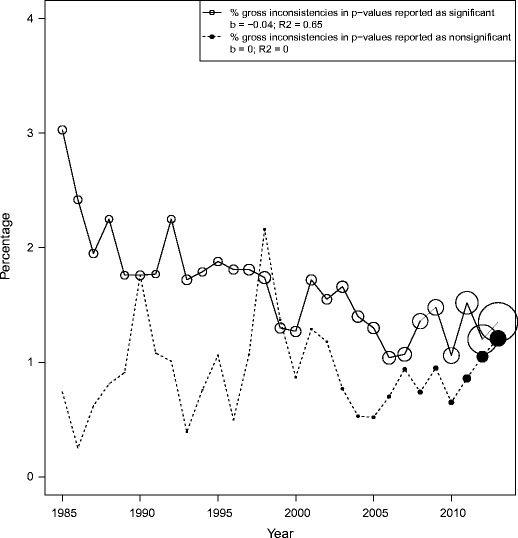
The percentage of gross inconsistencies in *p*-values reported as significant (solid line) and nonsignificant (dotted line), over the years, averaged over journals. The size of the open and solid circles represents the number of significant and nonsignificant *p*-values in that year, respectively

Figure 7 shows the prevalence of gross inconsistencies in significant (solid line) and nonsignificant (dotted line) *p*-values over time, averaged over all journals. The size of the circles represents the total number of significant (open circle) and nonsignificant (solid circle) *p*-values in that particular year. Note that we only have information for PS, FP, and PLOS since 2003, 2010, and 2004, respectively. The prevalence of gross inconsistencies in significant *p*-values seems to decline slightly over the years (*b* = −.04, R^2^ = .65). The prevalence of the gross inconsistencies in nonsignificant *p*-values does not show any change (*b* = .00, R^2^ = .00). In short, the potential systematic bias leading to more gross inconsistencies in significant results seems to be present in all journals except for FP, but there is no evidence that this bias is increasing over the years.

To investigate the consequence of these gross inconsistencies, we compared the percentage of significant results in the reported *p*-values with the percentage of significant results in the computed *p*-values. Averaged over all journals and years, 76.6 % of all reported *p*-values were significant. However, only 74.4 % of all computed *p*-values were significant, which means that the percentage of significant findings in the investigated literature is overestimated by 2.2 percentage points due to gross inconsistencies.

### Prevalence of inconsistencies as found by other studies

Our study can be considered a large replication of several previous studies (Bakker & Wicherts, 2011; Bakker & Wicherts, 2014; Berle & Starcevic, 2007; Caperos & Pardo, 2013; Garcia-Berthou & Alcaraz, 2004; Veldkamp et al., 2014; Wicherts et al., 2011). Table 2 shows the prevalence of inconsistent *p*-values as determined by our study and previous studies.

**Table 2:** Prevalence of inconsistencies in the current study and in earlier studies

Study	Field	No. of articles	No. of results	No. of inconsis-tencies	Gross inconsistencies (%)	Articles with at least one inconsistency (%)	Articles with at least one gross inconsistency (%)
Current study	Psychology	30,717	258,105	9.7	1.4	49.6^2^	12.9^2^
Garcia-Berthou and Alcaraz (2004)	Medical	44	244^4^	11.5	0.4	31.5	-
Berle and Starcevic (2007)	Psychiatry	345	5,464	14.3	-	10.1	2.6
Wicherts et al. (2011)	Psychology	49	1,148^1^	4.3	0.9	53.1	14.3
Bakker and Wicherts (2011)	Psychology	333	4,248^3^	11.9	1.3	45.4	12.4
Caperos and Pardo (2013)	Psychology	186	1,212^3^	12.2	2.3	48.0^2^	17.6^2^
Bakker and Wicherts (2014)	Psychology	153^5^	2,667	6.7	1.1	45.1	15.0
Veldkamp et al. (2014)	Psychology	697	8,105	10.6	0.8	63.0	20.5

^1^Only *t*, *F*, and *χ* ^*2*^ values with a *p* < .05

^2^Number of articles with at least one (gross) inconsistency/number of articles with null-hypothesis significance testing results

^3^Only included *t*, *F*, and *χ* ^*2*^ values

^4^Only exactly reported *p*-values

^5^Only articles with at least one completely reported *t* or *F* test with a reported *p*-value <.05

Table 2 shows that the estimated percentage of inconsistent results can vary considerably between studies, ranging from 4.3 % of the results (Wicherts et al., 2011) to 14.3 % of the results (Berle & Starcevic, 2007). The median rate of inconsistent results is 11.1 % (1.4 percentage points higher than the 9.7 % in the current study). The percentage of gross inconsistencies ranged from .4 % (Garcia-Berthou & Alcaraz, 2004) to 2.3 % (Caperos & Pardo, 2013), with a median of 1.1 % (.3 percentage points lower than the 1.4 % found in the current study). The percentage of articles with at least one inconsistency ranged from as low as 10.1 % (Berle & Starcevic, 2007) to as high as 63.0 % (Veldkamp et al., 2014), with a median of 46.7 % (2.9 percentage points lower than the estimated 49.6 % in the current study). Finally, the lowest percentage of articles with at least one gross inconsistency is 2.6 % (Berle & Starcevic, 2007) and the highest is 20.5 % (Veldkamp et al., 2014), with a median of 14.3 % (1.4 percentage points higher than the 12.9 % found in the current study).

Some of the differences in prevalences could be caused by differences in inclusion criteria. For instance, Bakker and Wicherts (2011) included only *t*, *F*, and *χ* ^*2*^ values; Wicherts et al. (2011) included only *t*, *F*, and *χ* ^*2*^ values of which the reported *p*-value was smaller than .05; Berle and Starcevic (2007) included only exactly reported *p*-values; Bakker and Wicherts (2014) only included completely reported *t* and *F* values. Furthermore, two studies evaluated *p*-values in the medical field (Garcia-Berthou & Alcaraz, 2004) and in psychiatry (Berle & Starcevic, 2007) instead of in psychology. Lastly, there can be differences in which *p*-values are counted as inconsistent. For instance, the current study counts *p* = .000 as incorrect, whereas this was not the case in for example Wicherts et al. (2011; see also Appendix A).

Based on Table 2 we conclude that our study corroborates earlier findings. The prevalence of reporting inconsistencies is high: almost all studies find that roughly one in ten results is erroneously reported. Even though the percentage of results that is grossly inconsistent is lower, the studies show that a substantial percentage of published articles contain at least one gross inconsistency, which is reason for concern.

## Discussion

In this paper we investigated the prevalence of reporting errors in eight major journals in psychology using the automated R package statcheck (Epskamp & Nuijten, 2015). Over half of the articles in the six flagship journals reported NHST results that statcheck was able to retrieve. Notwithstanding the many debates on the downsides of NHST (see e.g., Fidler & Cumming, 2005; Wagenmakers, 2007), the use of NHST in psychology appears to have increased from 1985 to 2013 (see Figs. 1 and 2), although this increase can also reflect an increase in adherence to APA reporting standards. Our findings show that in general the prevalence of reporting inconsistencies in six flagship psychology journals is substantial. Roughly half of all articles with NHST results contained at least one inconsistency and about 13 % contained a gross inconsistency that may have affected the statistical conclusion. At the level of individual *p*-values we found that on average 10.6 % of the *p*-values in an article were inconsistent, whereas 1.6 % of the *p*-values were grossly inconsistent.

Contrary to what one would expect based on the suggestion that QRPs have been on the rise (Leggett et al., 2013), we found no general increase in the prevalence of inconsistent *p*-values in the studied journals from 1985 to 2013. When focusing on inconsistencies at the article level, we only found an increase in the percentage of articles with NHST results that showed at least one inconsistency for JEPG and JPSP. Note this was associated with clear increases in the number of reported NHST results per article in these journals. Furthermore, we did not find an increase in gross inconsistencies in any of the journals. If anything, we saw that the prevalence of articles with gross inconsistencies has been decreasing since 1985, albeit only slightly. We also found no increase in the prevalence of gross inconsistencies in *p*-values that were reported as significant as compared to gross inconsistencies in *p*-values reported as nonsignificant. This is at odds with the notion that QRPs in general and reporting errors in particular have been increasing in the last decades. On the other hand, the stability or decrease in reporting errors is in line with research showing no trend in the proportion of published errata, which implies that there is also no trend in the proportion of articles with (reporting) errors (Fanelli, 2013).

Furthermore, we found no evidence that inconsistencies are more prevalent in JPSP than in other journals. The (gross) inconsistency rate was not the highest in JPSP. The prevalence of (gross) inconsistencies has been declining in JPSP, as it did in other journals. We did find that JPSP showed a higher prevalence of articles with at least one inconsistency than other journals, but this was associated with the higher number of NSHT results per article in JPSP. Hence our findings are not in line with the previous findings that JPSP shows a higher (increase in) inconsistency rate (Leggett et al., 2013). Since statcheck cannot distinguish between *p*-values pertaining to core hypotheses and *p*-values pertaining to, for example, manipulation checks, it is hard to interpret the differences in inconsistencies between fields and the implications of these differences. To warrant such a conclusion the inconsistencies would have to be manually analyzed within the context of the papers containing the inconsistencies.

We also found that gross inconsistencies are more prevalent in *p*-values reported as significant than in *p*-values reported as nonsignificant. This could suggest a systematic bias favoring significant results, potentially leading to an excess of false positives in the literature. The higher prevalence of gross inconsistencies in significant *p*-values versus nonsignificant *p*-values was highest in JCCP, JAP, and JPSP, and lowest in PLOS and FP. Note again that we do not know the hypotheses underlying these *p*-values. It is possible that in some cases a nonsignificant *p*-value would be in line with a hypothesis and thus in line with the researcher’s predictions. Our data do not speak to the causes of this over-representation of significant results. Perhaps these *p*-values are intentionally rounded down (a practice that 20 % of the surveyed psychological researchers admitted to; John et al., 2012) to convince the reviewers and other readers of an effect. Or perhaps researchers fail to double check significantly reported *p*-values, because they are in line with their expectations, hence leaving such reporting errors more likely to remain undetected. It is also possible that the cause of the over-representation of falsely significant results lies with publication bias: perhaps researchers report significant *p*-values as nonsignificant just as often as vice versa, but in the process of publication, only the (accidentally) significant *p*-values get published.

There are two main limitations in our study. Firstly, by using the automated procedure statcheck to detect reporting inconsistencies, our sample did not include NHST results that were not reported exactly according to APA format or results reported in tables. However, based on the validity study and on earlier results (Bakker & Wicherts, 2011), we conclude that there does not seem to be a difference in the prevalence of reporting inconsistencies between results in APA format and results that are not exactly in APA format (see Appendix A). The validity study did suggest, however, that statcheck might slightly overestimate the number of inconsistencies. One reason could be that statcheck cannot correctly evaluate *p*-values that were adjusted for multiple testing. However, we found that these adjustments are rarely used. Notably, the term “Bonferroni” was mentioned in a meager 0.3 % of the 30,717 papers. This finding is interesting in itself; with a median number of 11 NHST results per paper, most papers report multiple *p*-values. Without any correction for multiple testing, this suggests that overall Type I error rates in the eight psychology journals are already higher than the nominal level of .05. Nevertheless, the effect of adjustments of *p*-values on the error estimates from statcheck is expected to be small. We therefore conclude that, as long as the results are interpreted with care, statcheck provides a good method to analyze vast amounts of literature to locate reporting inconsistencies. Future developments of statcheck could focus on taking into account corrections for multiple testing and results reported in tables or with effect sizes reported between the test statistic and *p*-value.

The second limitation of our study is that we chose to limit our sample to only a selection of flagship journals from several sub disciplines of psychology. It is possible that the prevalence of inconsistencies in these journals is not representative for the psychological literature. For instance, it has been found that journals with lower impact factors have a higher prevalence of reporting inconsistencies than high impact journals (Bakker & Wicherts, 2011). In this study we avoid conclusions about psychology in general, but treat the APA-reported NHST results in the full text of the articles from journals we selected as the population of interest (which made statistical inference superfluous). All conclusions in this paper therefore hold for the APA-reported NHST results in the eight selected journals. Nevertheless, the relatively high impact factors of these journals attest to the relevance of the current study.

There are several possible solutions to the problem of reporting inconsistencies. Firstly, researchers can check their own papers before submitting, either by hand or with the R package statcheck. Editors and reviewers could also make use of statcheck to quickly flag possible reporting inconsistencies in a submission, after which the flagged results can be checked by hand. This should reduce erroneous conclusions caused by gross inconsistencies. Checking articles with statcheck can also prevent such inconsistencies from distorting meta-analyses or analyses of *p*-value distributions (Simonsohn et al., 2014; Van Assen et al., 2014). This solution would be in line with the notion of Analytic Review (Sakaluk, Williams, & Biernat, 2014), in which a reviewer receives the data file and syntax of a manuscript to check if the reported analyses were actually conducted and reported correctly. One of the main concerns about Analytic Review is that it would take reviewers a lot of additional work. The use of statcheck in Analytic Review could reduce this workload substantially.

Secondly, the prevalence of inconsistencies might decrease if co-authors check each other’s work, a so-called “co-pilot model” (Wicherts, 2011). In recent research (Veldkamp et al., 2014) this idea has been investigated by relating the probability that a *p*-value was inconsistent to six different co-piloting activities (e.g., multiple authors conducting the statistical analyses). Veldkamp et al. did not find direct evidence for a relation between co-piloting and reduced prevalence of reporting errors. However, the investigated co-pilot activities did not explicitly include the actual checking of each other’s *p*-values, hence we do not rule out the possibility that reporting errors would decrease if co-authors double checked *p*-values.

Thirdly, it has been found that reporting errors are related to reluctance to share data (Wicherts et al., 2011). Although any causal relation cannot be established, a solution might be to require open data by default, allowing exceptions only when explicit reasons are available for not sharing. Subsequently, researchers know their data could be checked and may feel inclined to double check the *Results* section before publishing the paper. Besides a possible reduction in reporting errors, sharing data has many other advantages. Sharing data for instance facilitates aggregating data for better effect size estimates, enable reanalyzing published articles, and increase credibility of scientific findings (see also Nosek, Spies, & Motyl, 2012; Sakaluk et al., 2014; Wicherts, 2013; Wicherts & Bakker, 2012). The APA already requires data to be available for verification purposes (American Psychological Association, 2010, p. 240), many journals explicitly encourage data sharing in their policies, and the journal Psychological Science has started to award badges to papers of which the data are publicly available. Despite these policies and encouragements, raw data are still rarely available (Alsheikh-Ali, Qureshi, Al-Mallah, & Ioannidis, 2011). One objection that has been raised is that due to privacy concerns data cannot be made publicly available (see e.g., Finkel, Eastwick, & Reis, 2015). Even though this can be a legitimate concern for some studies with particularly sensitive data, these are exceptions; the data of most psychology studies could be published without risks (Nosek et al., 2012).

To find a successful solution to the substantial prevalence of reporting errors, more research is needed on how reporting errors arise. It is important to know whether reporting inconsistencies are mere sloppiness or whether they are intentional. We found that the large majority of inconsistencies were not gross inconsistencies around *p* = .05, but inconsistencies that did not directly influence any statistical conclusion. Rounding down a *p*-value of, say, .38 down to .37 does not seem to be in the direct interest of the researcher, suggesting that the majority of inconsistencies are accidental. On the other hand, we did find that the large majority of grossly inconsistent *p*-values were nonsignificant *p*-values that were presented as significant, instead of vice versa. This seems to indicate a systematic bias that causes an over-representation of significant results in the literature. Whatever the cause of this over-representation might be, there seems to be too much focus on getting “perfect,” significant results (see also Giner-Sorolla, 2012). Considering that the ubiquitous significance level of .05 is arbitrary, and that there is a vast amount of critique on NHST in general (see e.g., Cohen, 1994; Fidler & Cumming, 2005; Krueger, 2001; Rozeboom, 1960; Wagenmakers, 2007), it should be clear that it is more important that *p*-values are accurately reported than that they are below .05.

There are many more interesting aspects of the collected 258,105 *p*-values that could be investigated, but this is beyond the scope of this paper. In another paper, the nonsignificant test results from this dataset are investigated for false negatives (Hartgerink, van Assen, & Wicherts, 2015). Here a method is used to detect false negatives and the results indicate two out of three papers with nonsignificant test results might contain false-negative results. This is only one out of the many possibilities and we publicly share the anonymized data on our Open Science Framework page (https://osf.io/gdr4q/) to encourage further research.

Our study illustrates that science is done by humans, and humans easily make mistakes. However, the prevalence of inconsistent *p*-values in eight major journals in psychology has generally been stable over the years, or even declining. Hopefully, statcheck can contribute to further reducing the prevalence of reporting inconsistencies in psychology.

## Figures and Tables

**Fig. 8:**
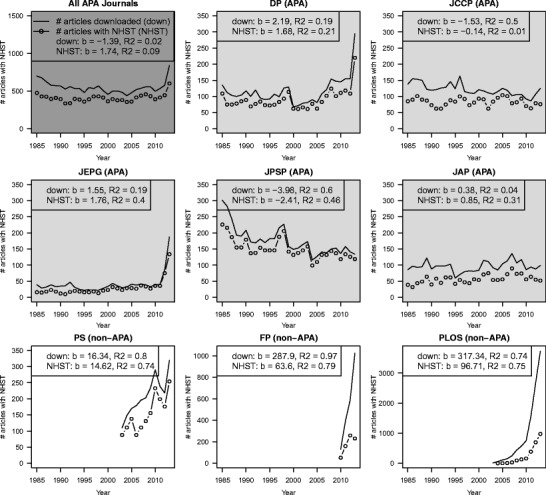
The total number of downloaded articles and the number of published articles that contain NHST results over the years, averaged over all American Psychological Association (APA) journals (*Developmental Psychology* (DP), *Journal of Consulting and Clinical Psychology* (JCCP), *Journal of Experimental Psychology: General* (JEPG), *Journal of Personality and Social Psychology* (JPSP), and *Journal of Applied Psychology* (JAP); dark gray panel), and split up per journal (light gray panels for the APA journals and white panels for the non-APA journals –*Psychological Science* (PS), *Frontiers in Psychology* (FP), and *Public Library of Science* (PLoS)). Note that the y-axes in the plot for All APA Journals, FP, and PLOS are different from the others and continue until 1,000, 1,050, and 3,750, respectively. The unstandardized regression coefficient ‘b’ and the coefficient of determination ‘R^2^’ of the linear trend are shown per journal for both the downloaded articles (down) as articles with null-hypothesis significance testing results (NHST) over the years

**Fig. 9:**
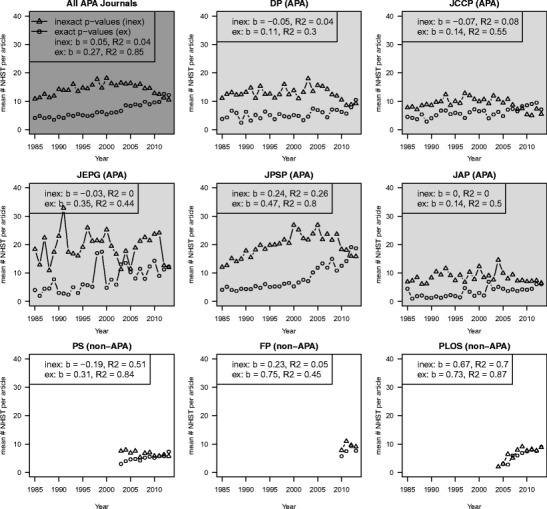
The average number of exact and inexact null-hypothesis significance testing (NHST) results per article over the years, averaged over all journals (grey panel), and split up by journal (white panels). The unstandardized regression coefficient ‘b’ and the coefficient of determination ‘R^2^’ of the linear trend are shown per journal for both exact (ex) as inexact (inex) p-values over the years. *APA* American Psychological Assocation, *DP* Developmental Psychology, *JCCP* Journal of Consulting and Clinical Psychology, JEPG Journal of Experimental Psychology: General , *JPSP* Journal of Personality and Social Psychology, *JAP* Journal of Applied Psychology, *PS* Psychological Science, *FP* Frontiers in Psychology, *PLoS* Public Library of Science

## Appendix A: Results validity check statcheck

Here we investigate the validity of the R program “statcheck” (Epskamp & Nuijten, 2015) by comparing the results of statcheck with the results of a study in which all statistics were manually retrieved, recalculated, and verified (Wicherts et al., 2011).

### Method

#### Sample

We used statcheck to scan the same 49 articles from the Journal of Experimental Psychology: Learning, Memory, and Cognition (JEP:LMC) and the Journal of Personality and Social Psychology (JPSP) that have been manually checked for reporting errors in Wicherts et al., who also double checked each reported error after it had been uncovered. The inclusion criteria for the statistical results to check for inconsistencies differed slightly between the study of Wicherts et al. and statcheck (see Table 3).

Both in Wicherts et al. and in this validity study only *p*-values smaller than .05 and only results from *t*, *F*, or χ^2^ tests were included. Wicherts et al. required the result to be reported completely. statcheck had the equivalent, but stricter criterion that the results had to be reported exactly according to American Psychological Association (APA) guidelines (in general: test statistic (degrees of freedom) =/</> …, *p* =/</>…). Furthermore, Wicherts et al. included all results reported in the text or a table in the *Results* section of an article. statcheck did not distinguish between different sections in a paper, but included all complete results in APA style. This, in practice, often excludes results reported in a table. Lastly, Wicherts et al. stated that they only evaluated results of NHST. statcheck did not explicitly have this criterion, but implicitly APA reported results of a *t*, *F*, or χ^2^ test will always be a NHST result.

#### Procedure

We ran statcheck on the 49 articles twice: once in default mode, and once with an automatic one-tailed test detection. The one-tailed test detection works as follows: if the words “one-tailed,” “one-sided,” or “directional” (with various spacing or punctuation) are mentioned in the article *and* a result is not an inconsistency if it is a one-tailed test, the result is counted as correct. From the complete statcheck results, we selected the cases in which the test statistic was *t*, *F*, or χ^2^, and in where *p* < .05.

### Results

#### Descriptives

Table 4 below shows the number of extracted statistics and the number of identified errors for both Wicherts et al., statcheck in default mode, and statcheck with the automatic one-tailed test detection.

Wicherts et al. extracted 1,148 results from the 49 articles, whereas statcheck extracted 775 results (67.5 %). Even though statcheck found fewer results, it found relatively more reporting errors (4.3 % of all results in Wicherts et al. vs. 9.0 % or 7.2 % of all results in statcheck, without or with one-tailed detection, respectively). In the next sections we will identify possible causes for these differences.

#### Explanations for discrepancies in the number of extracted statistics

We found that in 13 articles statcheck reported the exact same amount of statistics as Wicherts et al. In 23 articles Wicherts et al. found more statistics than statcheck, and in 13 articles statcheck found more results than Wicherts et al. Table 5 shows the explanations for these discrepancies.

Most of the results that statcheck missed were results that were not reported completely (e.g., results in tables) or not exactly according to the APA format (e.g., an effect size reported in between the test statistic and the *p*-value, or the results being reported in a sentence). Furthermore, one article in the sample of Wicherts et al. has been retracted since 2011, and we could not download it anymore; its 28 *p*-values were not included in the statcheck validity study.

Most of the results that were only included by statcheck but not by Wicherts et al. were results that were that were not reported in the *Results* section but in footnotes, in the *Methods* section, or in the *Discussion*. Wicherts et al. did not take these results into account; their explicit inclusion criterion was that the result had to be in the text or in a table in the *Results* section of a paper. statcheck could not make this distinction and included results independent from their location. Furthermore, Wicherts et al. did not include the two G^2^ statistics that statcheck counted as χ^2^ statistics. Statcheck also included an inexactly reported *F*-statistic that Wicherts et al. excluded, because it referred to multiple tests. Finally, we found two results that fitted their inclusion criteria, but were inadvertently not included by the Wicherts et al. sample.

#### Explanations for discrepancies in the number of identified inconsistencies

There were discrepancies in the number of (gross) inconsistencies that Wicherts et al. and statcheck found. Table 6 explains these inconsistencies in detail. In 13 cases Wicherts et al. found more errors than statcheck (with default options). However, all these cases were results that statcheck did not scan for one of the reasons mentioned above. There are no other cases in which Wicherts et al. found more errors. The use of default statcheck did not highlight any false negatives.

The default statcheck did, however, find 34 false positives (i.e., it marked results as inconsistent whereas Wicherts et al. did not). Closer inspection of these cases highlighted four main causes. Firstly, seven cases were not included in the sample of Wicherts et al. Secondly, seven of the results that statcheck classified as an error, but Wicherts et al. did not, were results in which the *p*-value was reported to be zero (*p* = .000). Wicherts et al. counted this as correct, in cases where rounding would indeed render *p* = .000. However, statcheck counts this as inconsistent, because a *p*-value this small should be reported as *p* < .001, but not as *p* = .000 (American Psychological Association, 2010, p. 114). Thirdly, there were 11 cases (in two articles) in which the *p*-value was inconsistent due to a Huyn-Feldt correction, which statcheck cannot take into account. Fourthly, there were nine cases in which the reported *p*-value was one-tailed and therefore twice as low as statcheck computed.

The discrepancies in the gross inconsistencies between the default statcheck and Wicherts et al. were due to seven one-tailed tests (see Table 7). Because of these one-tailed tests, statcheck gives an exaggerated image of how many inconsistencies there are in the literature. Therefore, we also inspect the results of statcheck with the one-tailed test detection.

When statcheck uses the one-tailed test detection all but one one-tailed test previously marked as inconsistent are now categorized as correct (see Tables 6 and 7).^2^ The one-tailed test detection does result in six more false negatives, in which an inconsistent two-tailed test is counted as correct (see Table 6). Overall, statcheck now detected 56 inconsistencies in 775 *p*-values (7.2 %) and eight gross inconsistencies (1.0 %), which is closer to the inconsistency prevalence found by Wicherts et al. (4.3 % and .9 %, respectively) than without the one-tailed test detection. In sum, statcheck performs better with the one-tailed test detection.

#### Inter-rater reliability manual versus statcheck

We also calculated the inter-rater reliability between the manual coding of inconsistencies and gross inconsistencies in Wicherts et al. and the automatic coding in statcheck. We distinguished between three different scenarios: in the first statcheck ran in default mode (without one-tailed test detection), in the second the automatic one-tailed test detection in statcheck was switched on, and in the last we ran statcheck with the automatic one-tailed test detection and we excluded cases in which *p* was reported as *p* = .000, since this was not counted as an inconsistency in Wicherts et al., but statcheck is intentionally programmed to see this as an inconsistency (since *p* cannot be zero and it should have been reported as *p* < .001). In all three scenarios we only included *p*-values that were rated both by Wicherts et al. and statcheck.

Table 8 shows the inter-rater reliabilities for the inconsistencies and gross inconsistencies in the three scenarios. If statcheck is ran without one-tailed test detection, Cohen’s kappa for the inconsistencies is .71 and for the gross inconsistencies .74. If we turn on the automatic one-tailed test detection, Cohen’s kappa for the gross inconsistencies increases to .89, but it slightly decreases for the inconsistencies to .69. Note, however, there are fewer *p*-values that statcheck wrongly marked as inconsistent with the one-tailed test detection (see Table 5). When both the one-tailed detection is switched on and we exclude cases in which *p* is reported as *p* = .000, Cohen’s kappa for the inconsistencies increases to .76, and remains at .89 for the gross inconsistencies.

### Discussion

In this validity check we compared the results of Wicherts et al. (2011) with the results of the default version of statcheck and statcheck with automatic one-tailed test detection. The results show that statcheck extracted 67.5 % of the manually retrieved results. The main reason for this is that statcheck could not read results that were not reported completely or not in APA style. Even though statcheck included fewer results than Wicherts et al., it found more inconsistencies. These inconsistencies were mainly one-tailed tests that were counted as inconsistent. Specifically, Wicherts et al. found 49 of the 1,148 results (4.3 %) to be inconsistent and ten to be grossly inconsistent (.9 %), whereas statcheck found 70 of the 775 results (9.0 %) to be inconsistent and 17 (2.2 %) to be grossly inconsistent. In other words, statcheck found an inconsistency rate that was 4.7 percentage points higher than the one found in a manual search and a gross inconsistency rate that is 1.3 percentage point higher. The inter-rater reliability for inconsistencies was .71 and for gross inconsistencies .74.

When statcheck was run with automatic one-tailed test detection, it still found more errors than did Wicherts et al. but the difference was smaller. Now statcheck found 56 of 775 results (7.2 %) to be inconsistent and eight results (1.0 %) to be grossly inconsistent. That means that with automatic one-tailed test detection statcheck found an inconsistency rate of only 2.9 percentage points higher than the one found in a manual search and a gross inconsistency rate of .1 percentage point higher. The inter-rater reliability for gross inconsistencies was as high as .89, but decreased slightly for inconsistencies to .69. However, since there are fewer *p*-values wrongly marked as inconsistent with the automatic one-tailed test detection, we advise users to use this option when searching for reporting inconsistencies.

The main limitation of statcheck is that it seems to give an overestimation of the number of inconsistencies in a sample. A large part of these false positives were due to the conscious choice to count *p* = .000 as inconsistent. If we exclude these cases, the inter-rater reliability for inconsistencies goes up to .76, and remains .89 for gross inconsistencies (with automatic one-tailed test detection).Furthermore, the false positives caused by one-tailed tests are mostly solved by statcheck’s one-tailed test detection. That leaves only the false positives due to *p*-values adjusted for multiple testing, eventually resulting in only a slight overestimation of the inconsistencies. Herein lies a possibility for future improvement of the program.

In conclusion, since statcheck slightly overestimated the prevalence of inconsistencies in our study, its results should be interpreted with care. We also advise against using statcheck blindly to point out mistakes in a single article. The main two usages of statcheck are: (1) to give an overall indication of the prevalence of inconsistencies in a large amount of literature, and (2) to give a first indication of inconsistent *p*-values in a single article, after which the results should be checked by hand. The final verdict on whether a result is erroneous should be based on careful consideration by an expert.

## Appendix B: Additional Analyses

### Number of articles with null-hypothesis significance testing results

Figure 8 shows the number of articles that contain null-hypothesis significance testing (NHST) results over the years averaged over all American Psychological Association (APA) journals (*Developmental Psychology* (DP), *Journal of Consulting and Clinical Psychology* (JCCP), *Journal of Experimental Psychology: General* (JEPG), *Journal of Personality and Social Psychology* (JPSP), and *Journal of Applied Psychology* (JAP); dark gray panel) and split up per journal (light gray panels for the APA journals and white panels for the non-APA journals –*Psychological Science* (PS), *Frontiers in Psychology* (FP), and *Public Library of Science* (PLoS)). The number of articles with NHST results seems to remain relatively stable over the years in JCCP and JAP. JPSP has published fewer articles with NHST results over the years. In DP and JEPG the number of articles with NHST results increased over the years. The newer journals PS, FP, and especially PLOS show a steep increase in articles with NHST results in the last few years.

#### Number of exactly and inexactly reported *p*-values over the years

Besides the general prevalence of NHST results over the years, we were also interested in the prevalence of exactly reported *p*-values (*p* = …) and inexactly reported *p*-values (*p* </> …, or “ns”, which could be interpreted the same as *p* > .05).^3^ From the fourth edition of the APA Publication Manual onward (1994), researchers have been encouraged to report *p*-values exactly, so we expected to find an increase of exactly reported *p*-values.

We inspected the prevalence of exact and inexact *p*-values over time averaged over all APA journals (DP, JCCP, JEPG, JPSP, and JAP; dark gray panel in Fig. 9), and split up per journal (light gray panels for the APA journals and white panels for the non-APA journals in Fig. 9). The average number of exact *p*-values per article with NHST results increases for all journals. For all journals except JAP and PS the number of inexact *p*-values per article with NHST results increased, although the increase is less steep than for exact *p*-values.

**Table 3:** Inclusion criteria for the statistical results to check for inconsistencies in Wicherts et al. (2011) and statcheck

Wicherts et al.	statcheck
p < .05	p < .05
*t*, *F*, χ^2^	*t*, *F*, χ^2^
Complete (test statistic, df, p)	APA (test statistic, df, p)
Main text or table in result section	-
NHST	-

*df* degrees of freedom, *APA* American Psychological Association, *NHST* null-hypothesis significance testing

**Table 4:** The number of extracted statistics and the number of identified errors for both Wicherts et al. and statcheck (with automatic one-tailed test detection)

	Wicherts et al.	statcheck	statcheck with one-tailed test detection
No. of articles	49	43	43
No. of results	1,148	775 (67.5 %)	775 (67.5 %)
No. of inconsistencies	49 (4.3 %)	70 (9.0 %)	56 (7.2 %)
No. of papers with at least one inconsistency	23 (46.9 %)	23 (53.5 %)^1^	21 (48.8 %)^1^
No. of gross inconsistencies	10 (0.9 %)	17 (2.3 %)	8 (1.0 %)
No. of papers with at least one gross inconsistency	7 (14.3 %)	10 (23.3 %)^1^	5 (11.6 %)^1^

^1^ Number of articles with at least one (gross) inconsistency/number of articles with null-hypothesis significance testing results

**Table 5:** Explanation of the discrepancies between the number of results that Wicherts et al. (2011) and statcheck extracted

	Type of discrepancy	No. of articles	No. of results	Example
More results extracted by Wicherts et al.	Value between test statistic and *p*-value	11	201	F1(1, 31) = 4.50, MSE = 22.013, p <.05
	Table (incomplete result)	8	150	
	Result in sentence	3	8	F(1, 15) = 19.9 and 5.16, p <.001 and p <.05, respectively
	Non-APA	5	49	F(1. 47) = 45.98, p <.01; F[1, 95] = 18.11, p <.001; F(l, 76) = 23.95, p <.001; no p value reported
	Article retracted	1	28	
More results extracted by statcheck	G^2^ statistic included as χ^2^ statistic	1	2	Δ G^2^(1) = 6.53, p =.011
	Footnote	12	31	
	Error Wicherts et al.: overlooked result	2	2	
	Inexact test statistic	1	1	
	Not in result section	9	27	Result in materials, procedure, discussion etc.
Total no. of extracted results Wicherts et al.		49	1148	
Total no. of extracted results statcheck		43	775	

**Table 6:** Explanation of the discrepancies between the number of inconsistencies found by Wicherts et al. (2011) and statcheck (with automatic one-tailed test detection)

	Category inconsistency	Statcheck		Statcheck with one-tailed test detection	
		No. of articles	No. of results	No. of articles	No. of results
More inconsistencies found by Wicherts et al.	Not scanned by statcheck	8	13	8	13
	Wrongly marked as one-tailed	0	0	3	6
More inconsistencies found by statcheck	*p* = .000 counted as incorrect	1	7	1	7
	One-tailed	4	9	1	1
	Not checked by Wicherts et al.	5	7	5	7
	Huyn-Feldt correction	2	11	2	11
Total no. of inconsistencies Wicherts et al.			49		49
Total of inconsistencies statcheck			70		56

**Table 7:** Explanation of the discrepancies between the number of gross inconsistencies found by Wicherts et al. (2011) and statcheck (with automatic one-tailed test detection)

	Category gross inconsistency	Statcheck		Statcheck with one-tailed test detection	
		No. of articles	No. of results	No. of articles	No. of results
More gross inconsistencies found by Wicherts et al.	Wrongly marked as one-tailed	0	0	2	2
More gross inconsistencies found by statcheck	One-tailed	4	7	0	0
Total no. of gross inconsistencies Wicherts et al.			10		10
Total no. of gross inconsistencies statcheck			17		8

**Table 8:** The inter-rater reliability expressed in Cohen’s kappa between the manual coding in Wicherts et al. (2011) and the automatic coding in statcheck without or with automatic one-tailed detection, and with and without exclusion of *p* = .000

	Inconsistencies	Gross inconsistencies
No automatic one-tailed test detection	.71	.74
Automatic one-tailed test detection	.69	.89
Automatic one-tailed test detection and exclude *p* = .000	.76	.89

## Footnotes

1 We note there is a minor difference in the number of search results from the webpage and the package due to default specifications in the rplos package. See also https://github.com/ropensci/rplos/issues/75
